# Plasma biomarkers for Alzheimer’s Disease in relation to neuropathology and cognitive change

**DOI:** 10.1007/s00401-022-02408-5

**Published:** 2022-02-23

**Authors:** Denis S. Smirnov, Nicholas J. Ashton, Kaj Blennow, Henrik Zetterberg, Joel Simrén, Juan Lantero-Rodriguez, Thomas K. Karikari, Annie Hiniker, Robert A. Rissman, David P. Salmon, Douglas Galasko

**Affiliations:** 1 University of California, San Diego and Shiley-Marcos Alzheimer’s Disease Research Center, La Jolla, CA USA; 2 Institute of Neuroscience and Physiology, Department of Psychiatry and Neurochemistry, Sahlgrenska Academy, University of Gothenburg, Mölndal, Sweden; 3 Institute of Psychiatry, Psychology and Neuroscience, King’s College London, Maurice Wohl Clinical Neuroscience Institute, London, UK; 4 NIHR Biomedical Research Centre for Mental Health and Biomedical Research Unit for Dementia at South London and Maudsley NHS Foundation, London, UK; 5 Wallenberg Centre for Molecular and Translational Medicine, Department of Psychiatry and Neurochemistry, Institute of Neuroscience and Physiology, Sahlgrenska Academy at the University of Gothenburg, Gothenburg, Sweden; 6 Clinical Neurochemistry Laboratory, Sahlgrenska University Hospital, Mölndal, Sweden; 7 Department of Neurodegenerative Disease, UCL Institute of Neurology, Queen Square, London, UK; 8 UK Dementia Research Institute, London, UK; 9 Hong Kong Center for Neurodegenerative Diseases, Hong Kong, China; 10 Department of Psychiatry, University of Pittsburgh, Pittsburgh, PA USA; 11 Department of Neurosciences, UC San Diego, 9500 Gilman Drive, La Jolla, CA 92093-0624 USA

**Keywords:** Biomarker, Alzheimer’s Disease, Neuropathology, Plasma, Dementia

## Abstract

Plasma biomarkers related to amyloid, tau, and neurodegeneration (ATN) show great promise for identifying these pathological features of Alzheimer’s Disease (AD) as shown by recent clinical studies and selected autopsy studies. We have evaluated ATN plasma biomarkers in a series of 312 well-characterized longitudinally followed research subjects with plasma available within 5 years or less before autopsy and examined these biomarkers in relation to a spectrum of AD and related pathologies. Plasma Aβ42, Aβ40, total Tau, P-tau181, P-tau231 and neurofilament light (NfL) were measured using Single molecule array (Simoa) assays. Neuropathological findings were assessed using standard research protocols. Comparing plasma biomarkers with pathology diagnoses and ratings, we found that P-tau181 (AUC = 0.856) and P-tau231 (AUC = 0.773) showed the strongest overall sensitivity and specificity for AD neuropathological change (ADNC). Plasma P-tau231 showed increases at earlier ADNC stages than other biomarkers. Plasma Aβ42/40 was decreased in relation to amyloid and AD pathology, with modest diagnostic accuracy (AUC = 0.601). NfL was increased in non-AD cases and in a subset of those with ADNC. Plasma biomarkers did not show changes in Lewy body disease (LBD), hippocampal sclerosis of aging (HS) or limbic-predominant age-related TDP-43 encephalopathy (LATE) unless ADNC was present. Higher levels of P-tau181, 231 and NfL predicted faster cognitive decline, as early as 10 years prior to autopsy, even among people with normal cognition or mild cognitive impairment. These results support plasma P-tau181 and 231 as diagnostic biomarkers related to ADNC that also can help to predict future cognitive decline, even in predementia stages. Although NfL was not consistently increased in plasma in AD and shows increases in several neurological disorders, it had utility to predict cognitive decline. Plasma Aβ42/40 as measured in this study was a relatively weak predictor of amyloid pathology, and different assay methods may be needed to improve on this. Additional plasma biomarkers are needed to detect the presence and impact of LBD and LATE pathology.

**Supplementary Information:**

The online version contains supplementary material available at 10.1007/s00401-022-02408-5.

## Introduction

Pathological processes underlying Alzheimer’s Disease (AD) include the aggregation of *β*-amyloid (A) and tau (T) proteins (forming plaques and tangles, respectively) and progressive neurodegeneration (N), which can be combined into an ATN framework for diagnosis and staging of disease [15]. Exciting advances have occurred in fluid biomarkers for AD and related disorders—sensitive assays for protein biomarkers in plasma may enable aspects of the ATN framework to be applied in a convenient and cost-effective manner [12]. Validation studies of plasma amyloid beta 42 (Aβ42 and the ratio of Aβ42/Aβ40), tau, and forms of phosphorylated tau (P-tau) that were conducted primarily in relation to clinical diagnoses and positron emission tomography (PET) imaging biomarkers and/or cerebrospinal fluid (CSF) biomarkers have shown high sensitivity and specificity to detect AD-type amyloid and tau pathology [3, 17, 25, 31, 40]. Several studies have examined plasma biomarkers against neuropathology findings noted at autopsy and have found concordance between higher P-tau levels and tangle burden [3, 7, 20, 31]; a few studies have evaluated plasma Aβ42 and neurofilament light (NfL) against pathology [2, 7]. Studies to date, however, have been relatively small and have not examined biomarkers in relation to the heterogeneity of AD pathology. Patients with AD may vary with respect to amyloid (e.g., the degree of diffuse vs neuritic plaques; extent of cerebral amyloid angiopathy), and the co-occurrence of common neurodegenerative pathologies (*α*-synuclein and TDP-43 pathology) or vascular brain pathology.

Here, we examine the central plasma biomarkers that relate to the ATN framework in a large series of patients followed at a single research center who had neuropathologic evaluation of their brains at autopsy. We address questions of how well each biomarker distinguishes people with definite AD pathology from those with minimal or intermediate pathology, how these markers change longitudinally in plasma in relation to age and brain pathology, and whether co-pathology and different types of amyloid pathology influence these plasma biomarkers. Finally, we examine the relationship between plasma biomarkers and cognitive change.

## Materials and methods

### Participants and clinical methods

Participants were volunteers enrolled in the UCSD Shiley-Marcos Alzheimer’s Disease Research Center (ADRC) who underwent longitudinal annual assessments, were followed until death, and agreed to brain examination at autopsy. The research protocol was reviewed and approved by the human subject review board at the University of California, San Diego (UCSD). Informed consent was obtained from all patients or their caregivers consistent with California State law.

The comprehensive annual clinical assessment evaluates participants and uses information provided by knowledgeable informants, and includes medical and neurological history, mental status testing, assessment of psychiatric symptoms with the Neuropsychiatric Inventory (NPI), ratings of functional impairment, Clinical Dementia Rating (CDR) total score and its six subdomain scores (i.e., CDR sum of boxes), structured neurological examination, and neuropsychological assessment that is based on a comprehensive battery of cognitive tests that includes tests of global cognition (Mini-Mental State Examination [MMSE] and Dementia Rating Scale [DRS]) and tests of memory, language, attention, executive function and visuospatial abilities (at least two tests per domain) [9]. Based on each annual evaluation, participants received a research diagnosis at a consensus conference that determined an overall evaluation of cognition (normal, mild cognitive impairment [MCI; diagnosed following standard criteria], or dementia) [9]. In participants with MCI or dementia, one or more etiological diagnoses were assigned, following research criteria (e.g., AD, Dementia with Lewy Bodies [DLB], Parkinson’s Disease with Dementia, Frontotemporal degeneration [FTD], other dementias). A subset of 76 subjects in our study had a research-grade brain MRI within 24 months or less of one of the plasma biomarker analyses. We analyzed the ROC for MRI imaging parameters analyzed using Freesurfer version 5.3.0 and plasma biomarkers vs ADNC at autopsy in this subgroup.

Blood was drawn from participants every 2 years unless they were unable to come to the ADRC for their evaluation. For this study, we analyzed plasma samples from subjects who had detailed neuropathological evaluation and their last blood draw 5 years or less before death. We excluded participants if they had a known dominantly inherited mutation for AD (i.e., *PSEN1*, *PSEN2* or *APP* mutations), a family history of autosomal dominant AD, or a reported age at onset (estimated from clinical interviews with the patient and an informant) younger than 50. All data (clinical and plasma) were limited to the last 10 years prior to death. For this study, baseline visit refers to the earliest blood draw (within 10 years of death) and last visit refers to the last blood draw before death. All clinic visits from baseline were used to model changes in cognition.

### Pathology

Autopsy was performed using a standardized protocol [36]. Brains were divided sagittally and the left hemibrain was fixed in 10% buffered formalin while the right hemibrain was sectioned coronally and frozen at − 80 °C. The formalin-fixed left hemibrain was cut serially into 1 cm slices for paraffin embedding. Sections were taken and stained with hematoxylin and eosin (H&E) for histopathological examination from: middle frontal cortex (Brodmann areas 8/9), rostral superior temporal cortex, inferior parietal cortex, hippocampus (CA1–CA4 and dentate gyrus), entorhinal cortex, basal ganglia, midbrain with substantia nigra, pons with locus coeruleus, and cerebellar cortex with dentate nucleus. Lesions were evaluated in 10-µm-thick sections stained with thioflavin-S or in 5-µm-thick sections with immunohistochemical staining.

#### AD pathology

Neuritic plaques, diffuse plaques, and neurofibrillary tangles (NFT) were identified either with 1% thioflavin-S stains viewed with ultraviolet illumination and a 440 µm bandpass wavelength excitation filter, or with immunohistochemical staining using antibodies to Aβ (Ab 69D, rabbit polyclonal from Edward Koo, 1:1200) and PHF1 tau (from Albert Einstein SOM (courtesy Peter Davies), 1:600). Neuritic plaque density was estimated using methods recommended by CERAD [26], and Braak stage for NFT pathology was determined [6]. For more recent cases, pathological diagnosis of AD was made using NIA-AA consensus criteria for the postmortem diagnosis of AD, wherein Thal phase 4–5 (A3), Braak stage V–VI (B3), and moderate-to-severe neuritic plaque density (C2/3) corresponds to high AD neuropathologic change (ADNC) [27, 38]. We were missing Thal phase from many cases because this evaluation was implemented only after it had been proposed as part of the NIA-AA 2012 Guidelines [27]. For consistency across older and newer cases, we, therefore, used NIA-Reagan criteria to define High ADNC versus all other categories [13]. For biomarker-pathology comparisons, we evaluated comparisons with NIA-Reagan, Braak stage and CERAD stages separately. Cerebral Amyloid Angiopathy (CAA) was graded from 0 (absent) to 3 (severe) according to procedures described by the NACC Neuropathology Working Group [27].

*Non-AD pathology*. Lewy body pathology identified by H&E staining and immunostaining with antibodies against α-synuclein (phospho-synuclein 81A, from Virginia Lee, 1:15,000) was staged according to consensus Lewy body disease (LBD) guidelines, with modification as suggested in Montine et al. to simplify the number of brain regions examined, into “brainstem”, “limbic” (transitional), or “neocortical” subtypes [22]. Individuals with amygdala-predominant Lewy bodies were not included as concomitant LBD, given the low likelihood of a clinical diagnosis of LBD in this group [39]. TDP-43 pathology was identified by immunohistochemical staining (Proteintech #10782–2-AP polyclonal, 1:12,000). Staging according to Limbic-predominant Age-related TDP-43 Encephalopathy (LATE) consensus guidelines into “amygdala”, “hippocampal”, or “neocortical” stages was limited to a subset of autopsy cases processed after 2017 [29]. We expanded this series by performing immunohistochemical staining for TDP-43 on a hippocampus section on selected additional cases that allowed us to define the presence or absence of hippocampal LATE neuropathologic change to explore the association with plasma biomarkers. Hippocampal sclerosis (HS) was assessed on all cases and diagnosed when neuronal loss in the CA1 and subiculum, accompanied by gliosis, was out of proportion with the degree of AD pathology—about 80% of these cases stained positive for TDP-43 [36]. Vascular pathology was assessed by examining the brain for large arterial and lacunar infarcts, microinfarcts, and hemorrhages. Arteriolosclerosis, atherosclerosis of the circle of Willis, and amyloid angiopathy were each rated as “none”, “mild”, “moderate”, or “severe” using a semi-quantitative 4-point scale. A Low Pathology group was defined as Braak 0–II and absence of significant LBD, major vascular pathology, LATE-NC, HS or other neurodegenerative pathology.

### Plasma sampling, handling and biomarker measurement

Plasma was prepared following the UCSD Shiley-Marcos ADRC standard operating procedures. Blood was drawn from a forearm vein into EDTA citrate vacutainer tubes and centrifuged at 2000×*g* for 10 min at 4 °C in a tabletop centrifuge within 1 h or less of blood draw. Plasma was separated and aliquoted into 500 µL fractions into polypropylene cryotubes (VWR or Sarstedt), snap frozen and stored at − 80º until biomarker analyses were conducted.

Plasma biomarkers were measured using Single molecule array (Simoa) assays (Quanterix, Inc) for Aβ42 and Aβ40, total tau (Neurology 3-Plex A Advantage Kit #101995), P-tau181 (pTau-181 V2 Advantage Kit #103714), P-tau231 (University Gothenburg) [3] and NfL (NF-light™ Advantage Kit #103186) at the Clinical Neurochemistry Laboratory, University of Gothenburg, Sweden. Across 14 analytical runs, plasma biomarkers had a repeatability (Aβ42 = 9.2%; Aβ40 = 5.2%, total tau = 3.3%; P-tau181 = 7.1%; P-tau231 = 5.9%) and an intermediate precision (Aβ40 = 5.7%, total tau = 9.2%; P-tau181 = 8.1%) of < 10%. Aβ42 and P-tau231 had an intermediate precision of 10.8% and 12.1%, respectively.

Values below assay lower limit of detection were excluded from modeling (since we did not know the true value), but were included in ROC analyses since they truly represent a low value. Values for 5 outlier samples were excluded where all biomarkers were markedly different from all other plasma draws from the same subject. Each of the subjects had at least one other plasma sample for comparison. One NfL value of 1154 pg/mL in a pathologically confirmed FTD patient was omitted from plots for visualization purposes but was retained in all statistical analyses.

### Statistical analysis

Continuous demographic data were compared among pathologically confirmed groups using ANOVA followed by Tukey’s HSD post hoc test if significant. Categorical demographic variables were examined using a Chi-squared test, followed by pairwise Chi-squared tests for significant results, with adjustment of *p* values for multiple comparisons using the Benjamini and Hochberg false discovery rate method. Comparisons of biomarkers across pathologic groups or features were made using linear regressions with adjustment for age, sex, and the interval from blood draw to death. Pairwise comparisons between groups after covariate adjustment in these models were performed using the *lsmeans* package for R, with p value adjustment for multiple comparisons by Tukey’s method using the Studentized range distribution to maintain a family error rate of 0.05. We report both unadjusted *p* values and *p* values adjusted for multiple comparisons using Tukey’s method in On-line Resource tables. Only multiple comparison-adjusted values are reported in the main text and graphically displayed on figures. Receiver-operating curve (ROC) analysis was used to evaluate plasma biomarkers in relation to different degrees of AD pathology. For these ROC analyses, 95% confidence intervals for the area under the curve (AUC) were computed with 2000 stratified bootstrap replicates. Comparisons of the AUCs between biomarkers and their combinations were performed using DeLong’s test. Correlations between the several CSF biomarkers were examined in various pathologically defined subgroups (e.g., AD, non-AD) using Pearson or Spearman analysis, as appropriate. Longitudinal analyses of changes of biomarkers and cognition over time used linear mixed models, with covariates added for age, sex, interval from last visit to death, as well as each variable’s interaction with time. All models included random intercepts and slopes by participant. Models of cognition further included terms for the baseline performance, as well as its interaction with time. Statistical analyses were conducted with R version 4.0.3

## Results

Demographic, clinical and *APOE* genotype data for 312 subjects divided into pathologically defined groups are shown in Table 1. On average, this autopsy cohort was relatively old with a mean age at baseline > 74 years for all groups, although the age range at baseline was wide (from 52 to 100 years; 25th percentile 71 years and 75th percentile 82 years). Sex distribution was relatively well balanced across groups, with a small male predominance. There was a marked over-representation of males in the Other Pathology groups (expected due to the male predominance of LBD). Mean MMSE scores were consistent with mild dementia at baseline in groups with significant AD or Other Pathology and showed progressive decline over follow-up; mean MMSE and DRS scores in the Low Pathology group showed minimal change over the follow-up interval (Table 1). *APOE* ε4 allele frequencies were (as expected) higher in Intermediate and High ADNC groups than in Low Pathology and Other Pathology groups. The Other Pathology group primarily included patients with HS or LBD, and smaller numbers of patients with FTD, vascular pathology, or several uncommon pathologies. Table 2 shows a more detailed breakdown of neuropathology findings in the various groups. As expected, High ADNC, with or without other pathologies, was associated with the greatest severity of neuritic plaques, diffuse plaques, and CAA.

**Table 1:** Subject characteristics at final blood draw across neuropathology groups

	Low pathology	Intermediate ADNC	High ADNC	Other pathology	Intermediate ADNC + other	High ADNC+ other	ANOVA/Chi-Sq *p* value
*n*	29	19	124	45	29	66	
Age at baseline	83.6 ± 6.9	85.3 ± 7.1	74.8 ± 9.4	77.8 ± 8.3	79.1 ± 6.3	77.1 ± 7.2	**1.5 × 10**^**–8 b,c,e,f,g,i**^
Age at last plasma	86.8 ± 6.1	89 ± 5.9	77.3 ± 9.9	80.1 ± 8.9	81.3 ± 6.7	79.4 ± 7.6	**2.4 × 10**^**–9 b,c,e,f,g,h,i**^
Age at death	88.7 ± 6.2	91 ± 6.4	79.8 ± 9.6	81.9 ± 9.2	82.8 ± 6.9	81.8 ± 7.8	**2.0 × 10**^**–8 b,c,e,f,g,h,i**^
Last blood draw to death (years)	1.9 ± 1.2	2 ± 1.3	2.5 ± 1.4	1.9 ± 1.2	1.5 ± 1.1	2.4 ± 1.3	**7.5 × 10**^**–4 k,o**^
Female	14 (48%)	8 (42%)	47 (38%)	8 (18%)	11 (38%)	18 (27%)	0.06
Hispanic	5 (17%)	3 (16%)	10 (8%)	3 (7%)	3 (10%)	3 (5%)	0.39
Education (years)	14.8 ± 3.2	15.4 ± 5	15.2 ± 3.4	15.5 ± 3.3	15.6 ± 3.4	15.8 ± 2.7	0.83
APOE 0 e4 alleles	20 (69%)	9 (47%)	51 (41%)	29 (64%)	14 (48%)	26 (39%)	**0.021 **^**b,e,o**^
APOE 1 e4 allele	9 (31%)	10 (53%)	57 (46%)	14 (31%)	14 (48%)	31 (47%)	
APOE 2 e4 alleles	0 (0%)	0 (0%)	16 (13%)	2 (4%)	1 (3%)	9 (14%)	
Baseline MMSE	27.9 ± 2.1	25.1 ± 4.6	21.9 ± 6.1	24.1 ± 4.9	22.4 ± 5.8	21.9 ± 5.3	**1.7 × 10**^**–6 b,c,d,e**^
Baseline DRS	131.2 ± 10.3	121.2 ± 14.9	109.8 ± 23.7	116.9 ± 17.6	112.7 ± 18.9	112.9 ± 16.3	**1.8 × 10**^**–5 b,c,d,e**^
Baseline CDR-sb	3.7 ± 4.1	5.3 ± 4.5	6.3 ± 3.8	5.9 ± 4	5.6 ± 4.3	6.3 ± 3.6	0.20
Last MMSE	27.1 ± 3.4	24.2 ± 4.1	15.1 ± 7.7	22.4 ± 5.4	18.1 ± 6.7	16.8 ± 5.7	**1.1 × 10**^**–20 b,c,d,e,f,h,i,j,n**^
Last DRS	128 ± 13	121 ± 13.7	86.1 ± 31.9	105.7 ± 27.6	96.6 ± 30.9	89.1 ± 23.9	**1.1 × 10**^**–13 b,c,d,e,f,h,I,j,n**^
Last CDR-sb	2.8 ± 4.3	5.9 ± 5.1	10.9 ± 4.4	7.4 ± 5.1	8.2 ± 4.8	10.3 ± 4.5	**7.6 × 10**^**–15 b,c,d,e,f,I,j,k,n**^
Last clinical diagnosis: normal	18 (62%)	3 (16%)	1 (1%)	5 (11%)	0 (0%)	0 (0%)	**2.8 × 10**^**–28 a,b,c,d,e,f,i,j,n**^
MCI	4 (14%)	4 (21%)	1 (1%)	2 (4%)	2 (7%)	2 (3%)	**7.6 × 10**^**–4 b,f**^
AD/dementia	7 (24%)	12 (63%)	108 (87%)	16 (36%)	16 (55%)	51 (77%)	**3.4 × 10**^**–14 a,b,d,e,f,j,k,n,o**^
DLB/PDD	0 (0%)	0 (0%)	8 (6%)	21 (47%)	10 (34%)	11 (17%)	**6.8 × 10**^**–11 c,d,g,h,j,k,n**^
FTLD	0 (0%)	0 (0%)	5 (4%)	1 (2%)	1 (3%)	2 (3%)	0.84
Other**	0 (0%)	0 (0%)	1 (1%)	0 (0%)	0 (0%)	0 (0%)	0.91
# of blood draws	2.6 ± 1.6	2.5 ± 1.8	2.5 ± 1.3	2.4 ± 1.4	2.4 ± 1.6	2.3 ± 1.1	0.98
# of annual visits	6.3 ± 3.1	5.2 ± 2.7	4.5 ± 2.3	4.3 ± 2.6	4 ± 2.4	3.9 ± 2.3	**3.0 × 10**^**–4 b,c,d,e**^

Tukey HSD post hoc comparisons significant with adjusted *p* < 0.05, or pairwise Chi-square tests with Benjamin–Hochberg adjusted *p* < 0.05

Missing data: Hispanic (*n* = 1, < 1%), first MMSE (*n* = 5, 1%), first DRS (*n* = 8, *n*%), first CDR-sb (*n* = 31, 10%), last MMSE (*n* = 8, 2%), last DRS (*n* = 12, 4%), last CDR-sb (*n* = 12, 4%)

*MMSE* Mini-Mental State Exam, *DRS* Dementia Rating Scale, *CSR-sb* Clinical Dementia Rating-sum of boxes

**Other clinical diagnosis was vascular dementia (*n* = 1)

^a^Low Path vs Intermediate ADNC, ^b^Low Path vs High ADNC, ^c^Low Path vs Other Path, ^d^Low Path vs Intermediate ADNC + Other, ^e^Low Path vs High ADNC + Other, ^f^Intermediate ADNC vs High ADNC, ^g^Intermediate ADNC vs Other Path, ^h^Intermediate ADNC vs Intermediate ADNC + Other, ^i^Intermediate ADNC vs High ADNC + Other, ^j^High ADNC vs Other Path, ^k^High ADNC vs Intermediate ADNC + Other, ^l^High ADNC vs High ADNC + Other, ^m^Other Path vs Intermediate ADNC + Other, ^n^Other Path vs High ADNC + Other

^o^Intermediate ADNC + Other vs High ADNC + Other

**Table 2:** Neuropathology: staging and subtypes

	Low Pathology	Intermediate ADNC	High ADNC	Other Path	Intermediate ADNC + Other	High ADNC + Other
*n*	29	19	124	45	29	66
Neuritic plaques: sparse	13 (45%)	0 (0%)	0 (0%)	31 (69%)	0 (0%)	0 (0%)
Neuritic plaques: moderate	16 (55%)	16 (84%)	32 (26%)	12 (27%)	20 (69%)	21 (32%)
Neuritic plaques: frequent	0 (0%)	3 (16%)	92 (74%)	2 (4%)	9 (31%)	45 (68%)
Braak 0–II	27 (93%)	0 (0%)	0 (0%)	39 (87%)	0 (0%)	0 (0%)
Braak III–IV	2 (7%)	19 (100%)	0 (0%)	6 (13%)	29 (100%)	0 (0%)
Braak V–VI	0 (0%)	0 (0%)	124 (100%)	0 (0%)	0 (0%)	66 (100%)
NIA-Reagan: not/Low ADNC	29 (100%)	0 (0%)	0 (0%)	45 (100%)	0 (0%)	0 (0%)
NIA-Reagan: Intermediate ADNC	0 (0%)	19 (100%)	0 (0%)	0 (0%)	29 (100%)	0 (0%)
NIA-Reagan: High ADNC	0 (0%)	0 (0%)	124 (100%)	0 (0%)	0 (0%)	66 (100%)
Thal phase 0–2	2 (7%)	1 (5%)	0 (0%)	4 (9%)	0 (0%)	1 (2%)
Thal phase 3	0 (0%)	1 (5%)	0 (0%)	1 (2%)	0 (0%)	1 (2%)
Thal phase 4–5	1 (3%)	3 (16%)	50 (40%)	4 (9%)	10 (34%)	30 (45%)
CAA: none/mild	20 (69%)	7 (37%)	46 (37%)	34 (76%)	15 (52%)	22 (33%)
CAA: moderate	5 (17%)	8 (42%)	37 (30%)	8 (18%)	7 (24%)	23 (35%)
CAA: severe	4 (14%)	4 (21%)	41 (33%)	3 (7%)	7 (24%)	21 (32%)
Diffuse plaques: sparse	12 (41%)	0 (0%)	10 (8%)	26 (58%)	0 (0%)	2 (3%)
Diffuse plaques: moderate	12 (41%)	7 (37%)	19 (15%)	6 (13%)	10 (34%)	11 (17%)
Diffuse plaques: frequent	5 (17%)	12 (63%)	95 (77%)	13 (29%)	19 (66%)	53 (80%)
LBD: brainstem	0 (0%)	0 (0%)	0 (0%)	6 (13%)	3 (10%)	4 (6%)
LBD: limbic	0 (0%)	0 (0%)	0 (0%)	9 (20%)	1 (3%)	7 (11%)
LBD: neocortical	0 (0%)	0 (0%)	0 (0%)	17 (38%)	17 (59%)	31 (47%)
Hippocampal sclerosis	0 (0%)	0 (0%)	0 (0%)	14 (31%)	8 (28%)	28 (42%)
FTLD	0 (0%)	0 (0%)	0 (0%)	4 (9%)	1 (3%)	4 (6%)
Other Pathology**	0 (0%)	0 (0%)	0 (0%)	4 (9%)	0 (0%)	3 (5%)
Vascular including microinfarcts	11 (38%)	8 (42%)	24 (19%)	6 (13%)	4 (14%)	5 (8%)

Missing data: Thal phase (*n* = 196, 63%)

**Other Pathology: neurodegeneration with brain iron accumulation (*n* = 3), multiple sclerosis (*n* = 1), limbic microglial nodular encephalitis (*n* = 1), atypical tauopathy with degeneration of substantia nigra (*n* = 1), alcoholic brain degeneration (*n* = 1)

**Fig. 1:**
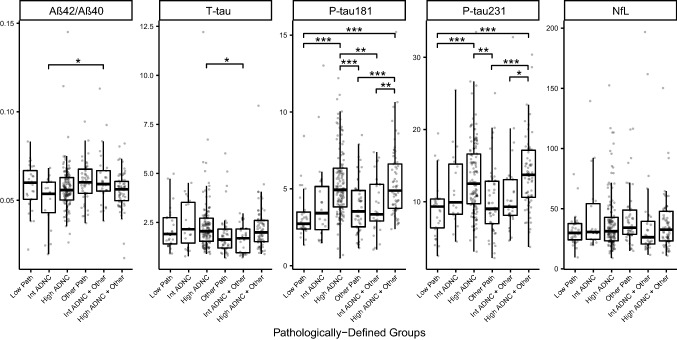
Plasma biomarkers at last blood draw by pathologic groups. Boxplots of the distributions of the plasma biomarkers from the blood draw closest to death by pathologic group. One NfL value of 1154 in FTLD patient was removed from plots for visualization but retained in statistical analyses. Effect sizes and both raw and multiple-comparisons adjusted *p* values are available in Supplementary Table 2, On-line Resource. Statistics for pairwise comparisons are corrected for multiple comparisons using Tukey’s method to maintain a family error rate of 0.05, and are graphically summarized as follows: *p < 0.05, **p < 0.01, ***p < 0.001

Plasma biomarkers at last blood draw for each pathologically defined subgroup are shown as box plots in Fig. 1, and summary data and detailed statistical comparisons between groups are shown in On-line Resource Tables 1 and 2. In analyses that adjusted for age, sex, and duration from last blood draw to death, the greatest differences were between High ADNC and Low Pathology for plasma P-tau181 (*p* = 9.19 × 10^–7^) and P-tau231 (*p* = 5.7 × 10^–7^). Plasma Aβ42 and Aβ42/Aβ40 (*p* = 0.014) showed a decrease in patients with High ADNC vs Low Pathology. Plasma total tau and NfL were not significantly altered in relation to ADNC. Figure 1 shows that in patients with Intermediate ADNC, P-tau181 and P-tau231 showed trends for increased levels compared to those with Low Pathology (P-tau181 showed more overlap).

We examined whether levels of plasma Aβ42 and Aβ42/Aβ40 were associated with different forms and measures of brain amyloid pathology that were assessed with ratings of diffuse plaques, neuritic plaques (CERAD), and presence and severity of CAA. We found that plasma Aβ42 and Aβ42/Aβ40 were decreased in people with moderate or severe diffuse plaques (vs. mild diffuse plaques), and plasma Aβ42 was slightly decreased in people with moderate CAA vs mild or absent CAA (Online Resource Fig. 1 and Online Resource Table 3). P-tau231 was increased in relation to severe CAA; however, severe CAA was more likely in people with higher neuritic plaque burden, and when we included neuritic plaque burden in the model, the association with CAA disappeared.

**Fig. 2:**
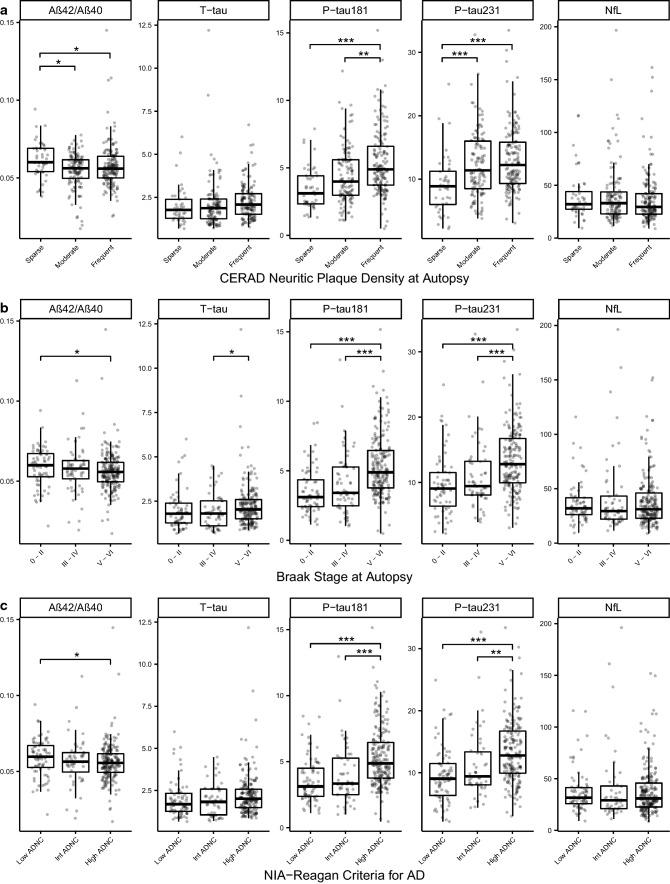
Plasma biomarkers in groups defined by different staging of AD Neuropathology. Boxplots of the distributions of the plasma biomarkers from the blood draw closest to death divided by **a** CERAD neuritic plaque density score, **b** Braak neurofibrillary tangle stage, and **c** NIA-Reagan Institute criteria stage of ADNC. One NfL value of 1154 in FTLD patient was removed from plots for visualization, but retained in statistical analyses. Effect sizes and both raw and multiple-comparisons adjusted *p* values are available in Supplementary Table 3, On-line Resource. Statistics for pairwise comparisons are corrected for multiple comparisons using Tukey’s method to maintain a family error rate of 0.05, and are graphically summarized as follows: *p < 0.05, **p < 0.01, ***p < 0.001

We next compared plasma biomarkers in people with various levels of ADNC defined by neuritic plaque burden, Braak stage (of tau pathology), or NIA-Reagan criteria (Fig. 2 and Online Resource Table 4). Aβ42/Aβ40 was decreased in association with severe neuritic plaque burden, but not across Braak stages. P-tau181 and P-tau231 were both increased in relation to neuritic plaque burden and Braak stage; however, P-tau181 increased across all three neuritic plaque grades, whereas P-tau231 increased sharply from no or sparse to mild neuritic plaques. This suggests that in plasma, P-tau231 may be altered earlier than P-tau181 in relation to neuritic plaque evolution. Both plasma P-tau biomarkers increased across Braak stages. Total tau was minimally increased in late-stage AD (Braak VI). NfL levels ranged widely and did not show a clear relationship to stages of AD pathology determined by these approaches.

**Fig. 3:**
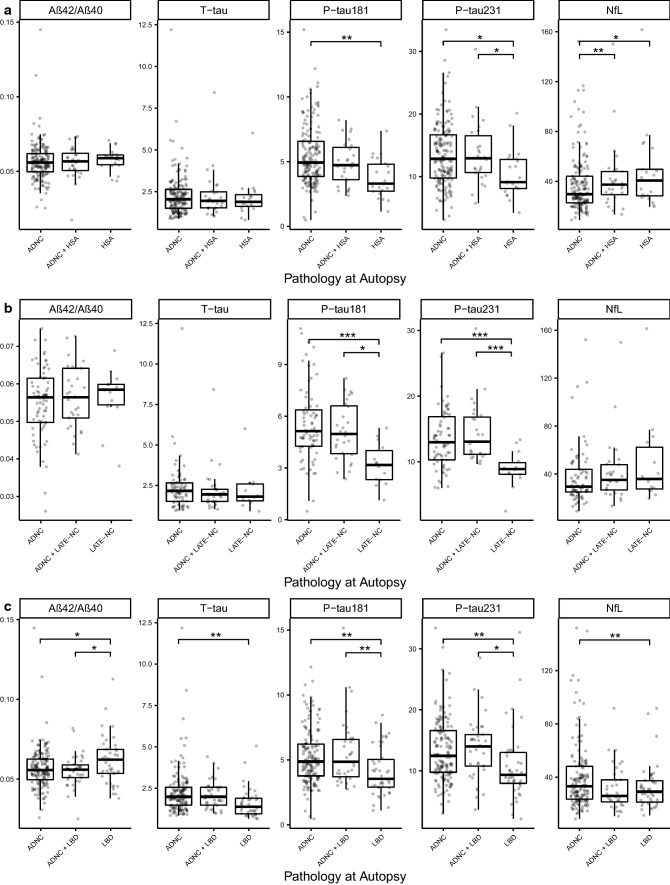
Plasma biomarkers in relation to Hippocampal sclerosis (HS), Limbic Age-related TDP-43 Encephalopathy (LATE), Lewy Body Disease (LBD) and ADNC. Boxplots of the distributions of the plasma biomarkers from the blood draw closest to death in individuals with High ADNC and/or other non-AD pathologies: **a** hippocampal sclerosis of aging defined as neuronal loss in the CA1 and subiculum out of proportion with the degree of AD pathology, **b** hippocampal staining positive for TDP-43 proteinopathy representing LATE neuropathologic changes (LATE-NC), and **c** Lewy body disease of the limbic (transitional) or neocortical (diffuse) type. These plots and analyses exclude participants who did not have either High ADNC or the non-AD pathology being assessed. TDP-43 immunostaining was available in a select subset of cases, with their demographic data available in Supplementary Table 4, On-line Resource. One NfL value of 1154 in a FTLD patient was removed from plots for visualization, but retained in statistical analyses. Effect sizes and both raw and multiple-comparisons adjusted *p* values are available in Supplementary Table 5, On-line Resource. Statistics for pairwise comparisons are corrected for multiple comparisons using Tukey’s method to maintain a family error rate of 0.05, and are graphically summarized as follows: *p < 0.05, **p < 0.01, ***p < 0.001

Within a subset of 76 participants who had a research-grade MRI within 24 months of an available plasma biomarker assessment, we compared ROC metrics for P-tau plasma biomarkers and for MRI measures of entorhinal thickness and hippocampal volume (adjusted for total intracranial volume). Results are shown in Fig. 3, Supplementary on-line resource. Plasma P-tau181 and 231 had higher AUC values than the MRI indices for distinguishing between High ADNC pathology and Other Pathology. A model including both entorhinal thickness and P-tau181 had only marginally better performance than P-tau181 alone.

We examined plasma biomarkers in other common age-related pathologies that can cause dementia, including two key pathologic features of LATE: HS which has been assessed on all cases) and abnormal hippocampal TDP-43 accumulation of LATE (which was assessed in a subset—see below), and LBD (defined as limbic or neocortical stages of α-synuclein pathology). We made the following comparisons: (1) HS without High ADNC, HS with High ADNC, and High ADNC without HS; LATE-NC without High ADNC, LATE-NC with High ADNC, and High ADNC without LATE-NC; and (3) LBD without High ADNC, LBD with High ADNC, and High ADNC without LBD. In HS, P-tau181 and P-tau231 were increased when High ADNC was present while NfL was slightly increased in HS with or without ADNC compared to ADNC alone (Fig. 3a, Online Resource Table 5). We fully staged LATE-NC in 64 cases and performed hippocampal TDP-43 immunohistochemistry in 52 additional cases selected to oversample HS and Intermediate ADNC. Summary data on this set of 116 cases are shown in Online Resource Table 6, and comparisons with plasma biomarkers in Fig. 3b. Relationships between higher levels of P-tau biomarkers in plasma and ADNC remained, whereas there were no significant associations between any of the plasma biomarkers and LATE-NC in the hippocampus. The main findings for LBD were that plasma Aβ42/40 was slightly decreased, and P-tau181 and P-tau231 significantly increased, if High ADNC was also present (Fig. 3c).

**Fig. 4:**
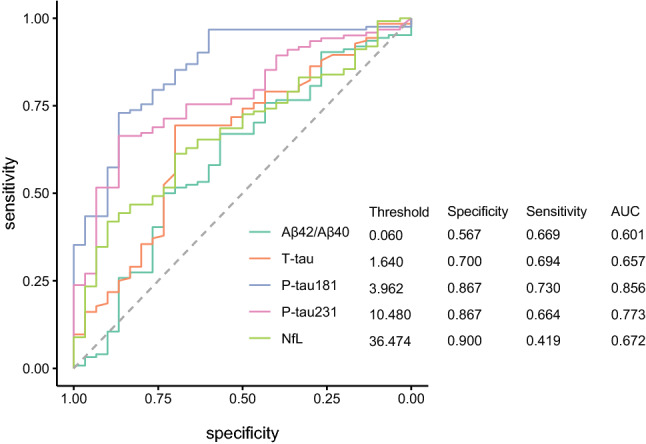
ROC analyses, comparing plasma biomarkers in the Low Pathology group vs the last blood draw in the High ADNC group. ROC curves and associated thresholds, specificities, sensitivities, and areas under the curve (AUCs) for the use of each plasma biomarker to distinguish patients who were classified as Low Pathology at autopsy from those who were classified as High ADNC. Because of the older age and stability of plasma biomarkers from baseline to last blood draw in the Low Pathology group, baseline plasma biomarkers in this group were compared to the final blood draw High ADNC group to achieve closer age matching

We performed ROC analyses to determine classification accuracy of the various plasma biomarkers using pathologically defined Low vs High ADNC groups as the “gold standard” to define cutoffs. Because of the older age and stability of plasma biomarkers from baseline to last blood draw in the low AD pathology group, we compared baseline plasma biomarkers in this group to last-blood-draw plasma biomarkers in the high likelihood AD group to achieve closer age matching. As shown in Fig. 4, the plasma biomarkers with the best classification accuracy were plasma P-tau181 followed by P-tau231. P-tau231 had relatively lower sensitivity than P-tau181 in these comparisons because it was increased in more people who died with Braak stage 0, I and II pathology than was P-tau181 (similar to relationships found in prior imaging studies where P-tau231 was increased as early as Braak stage II defined by Tau PET) [3].

**Fig. 5:**
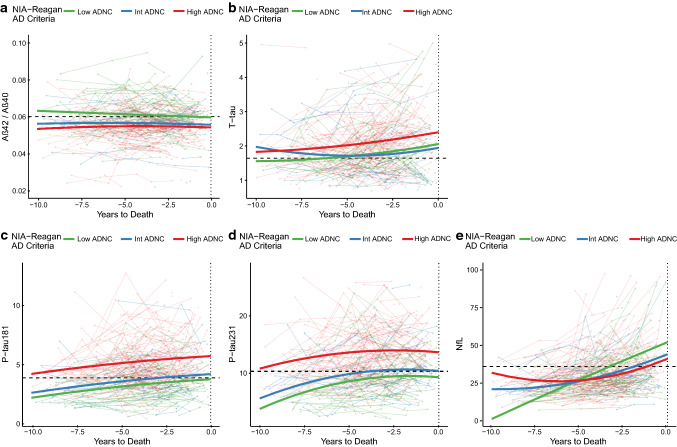
Longitudinal changes of plasma biomarkers in relation to ADNC. Longitudinal progression in biomarkers in the 10 years prior to death in all study participants were divided by their degree of ADNC. Horizontal dashed lines represent the thresholds derived from ROC analyses presented in Fig. 4. Thick lines represent predictions of the trajectories of the biomarkers for a demographically average participant, derived from mixed effects models with covariates added for age, sex, interval from last visit to death, as well as each variable’s interaction with time. All models included random intercepts and slopes by participant. A version of this figure and analysis excluding individuals with concomitant non-AD pathologies is available in Supplementary Fig. 2, On-line Resource

We modeled longitudinal changes in the various plasma biomarkers from baseline to death in relation to severity of ADNC (Fig. 5a–e shows these relationships for all cases and Online Resource Fig. 2a–e limited to people with ADNC). Of note, as early as 10 years prior to their death, participants who died with Intermediate or severe ADNC already showed changes in plasma biomarker levels relative to those with no or minimal ADNC (decreased plasma Aβ42/40 and increased P-tau181 and P-tau 231). The modeled mean levels of plasma P-tau181 and P-tau231 at baseline were already above the ROC cutoff in groups with Intermediate or severe ADNC and showed a trajectory of continued increase over time in all three severity groups (Low, Intermediate, High). Age of death and presence of an *APOE* ε4 allele did not modify these trajectories for plasma P-tau181 or P-tau231. Plasma NfL showed acceleration of change in people with moderate or high tau pathology, with the model crossing above the ROC diagnostic threshold about 4 years prior to death (Fig. 5b).

**Fig. 6:**
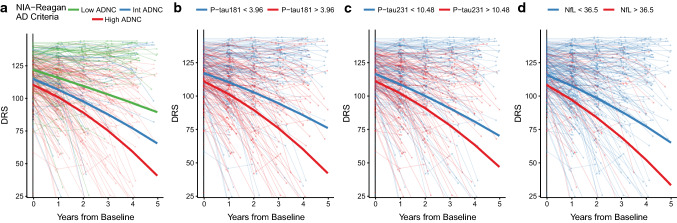
Plasma P-tau and NfL biomarkers and longitudinal cognitive change in relation to AD pathology (excluding FTLD, HS, LBD, and Other pathologies). Longitudinal progression on the Dementia Rating Scale (DRS) in the 5-year interval from baseline in study participants divided by their **a** degree of ADNC, or **b**–**d** baseline plasma biomarker levels, after excluding FTLD, HS, LBD, and other significant pathologies. Cutoffs for each biomarker are those derived from ROC analyses presented in Fig. 4. Thick lines represent predictions of the trajectories of the DRS for a demographically average participant, derived from mixed effects models with covariates added for age, sex, interval from last visit to death, education as well as each variable’s interaction with time. To account for different starting levels of impairment the baseline DRS score was included as an interaction with time. All models included random intercepts and slopes by participant. Statistics for Exponential time term by biomarker interaction: NIA-Reagan Low vs Int, *p* = 0.24, Low vs High *p* = 9.4 × 10^–12^, pTau181 *p* = 6.6 × 10^–7^, pTau231 *p* = 0.0022, NfL *p* = 0.021

**Fig. 7:**
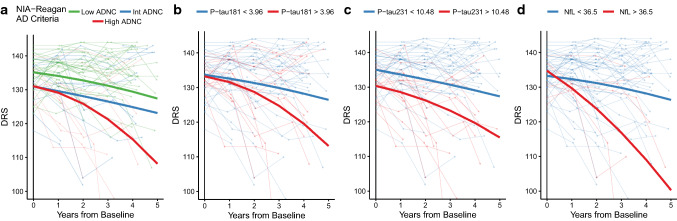
ADNC and baseline plasma P-tau and NfL biomarkers and longitudinal cognitive change in subjects with normal cognition or MCI at baseline. Longitudinal progression on the Dementia Rating Scale (DRS) in the 5-year interval from baseline in participants with normal cognition of mild cognitive impairment (MCI) divided by their **a** degree of ADNC, or **b**–**d** baseline plasma biomarker levels, after excluding FTLD, HS, LBD, and other significant pathologies. Cutoffs for each biomarker are those derived from ROC analyses presented in Fig. 4. Thick lines represent predictions of the trajectories of the DRS for a demographically average participant, derived from mixed effects models with covariates added for age, sex, interval from last visit to death, education as well as each variable’s interaction with time. To account for different starting levels of impairment the baseline DRS score was included as an interaction with time. All models included random intercepts and slopes by participant. Statistics for exponential time term by biomarker interaction: NIA-Reagan Low vs Int, *p* = 0.66, Low vs High *p* = 0.0074, pTau181 *p* = 0.0019, pTau231 *p* = 0.044, NfL *p* = 0.00524

Using the available longitudinal cognitive data, we analyzed changes in cognition in relation to plasma biomarkers and the burden of brain pathology. Figure 6a shows longitudinal change from baseline on total DRS score as a function of level of ADNC at death. As expected, people with eventual High ADNC had cognitive decline that was steeper and began earlier than those with eventual Intermediate or Low ADNC. When we modeled the relationship between DRS score decline and baseline plasma P-tau or NfL biomarkers in all subjects with AD pathology (Fig. 6b–d), we found that cognitive decline was steeper in patients with high (i.e., above our ROC-defined cutoffs) baseline plasma P-tau181 (> 3.64 pg/mL) or P-tau231 (> 10.5 pg/mL) levels than in those with low (below cutoff) levels of these plasma biomarkers. Although NfL had low sensitivity as a diagnostic biomarker, levels above the diagnostic cutoff strongly predicted faster cognitive decline. Analyses of baseline plasma Aβ42/40 did not strongly predict cognitive decline (data not shown). Similar results were found for analyses of predictors of progression on other clinical indices, e.g., CDR-sb (data not shown). In exploratory analyses of cognitive change in people who were assessed as having normal cognition or MCI at baseline, higher plasma P-tau181, 231 and NfL were associated with more rapid cognitive decline (Fig. 7a–d). Higher baseline P-tau231 levels showed weaker statistical significance, but levels at baseline were associated with lower DRS scores at baseline. Since NfL did not have strong associations with AD, LBD, HS, or LATE pathological changes, we assessed whether there were differences in vascular risk factors or other characteristics of the high vs low NfL subgroups. Those with high plasma NfL were more likely to have atrial fibrillation and history of TIA or stroke and were more likely to have microinfarcts and moderate-to-severe atherosclerosis of the circle of Willis (Table 3). These suggest that an additional burden of vascular risk and brain pathology may contribute to the increased levels of NfL in plasma associated with neurodegeneration.

**Table 3:** Vascular risk factors and neuropathological changes in the high and low plasma NfL subgroups

	NfL < 36.5	NfL > 36.5	*p* value
Number of participants	185	127	
Last age	78.8 ± 9.4	82 ± 8.5	**0.002**
Age at death	81.1 ± 9.3	84 ± 8.5	**0.005**
Smoking ever	38 (21%)	35 (28%)	0.18
History of cardiovascular disease	66 (36%)	54 (43%)	0.27
Atrial fibrillation	22 (12%)	29 (23%)	**0.02**
Diabetes mellitus	14 (8%)	9 (7%)	0.99
Hypertension	94 (51%)	78 (61%)	0.08
History of TIA or stroke	26 (14%)	29 (23%)	**0.04**
Pathology: cerebral infarct	16 (9%)	13 (10%)	0.78
Pathology: microinfarcts	7 (4%)	22 (17%)	**0.0001**
Pathology: atherosclerosis (moderate or severe)	58 (31%)	65 (51%)	**0.0004**

*p* < 0.05 in bold

Statistical comparisons by *t*-test for continuous variables and Chi-squared test for binary variables (presence/absence of risk factor or pathology)

**Fig. 8:**
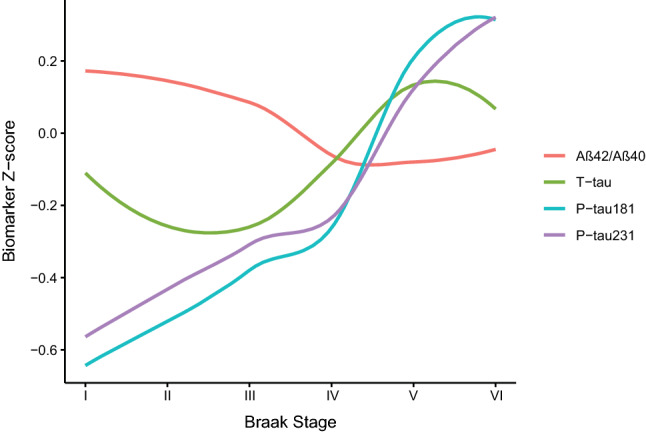
Plasma biomarker *Z*-scores and Braak stage. Local regression curves derived by locally estimated scatterplot smoothing of the *Z*-score of each plasma biomarker from the blood draw closest to death across Braak stage. The individual biomarker *Z*-scores were derived by setting the mean of the distribution to 0 and its standard deviation to 1

When we modeled trajectories of changes in plasma biomarkers across the spectrum of AD pathology (Fig. 8), we found that both P-tau231 and P-tau181 showed early increases, with P-tau231 changing slightly earlier relative to P-tau181. There was an increase in plasma levels of P-tau231 in some subjects without dementia who had low or intermediate tau pathology, suggesting that these early stages of AD regional pathology may be sufficient to cause the release of enough P-tau231 to be detectable in plasma. Total tau did not show a consistent pattern of longitudinal changes in relation to different grades of tau pathology.

## Discussion

Plasma ATN biomarkers show great promise for AD diagnosis and staging. Many studies have correlated plasma biomarkers with amyloid and tau PET imaging, CSF biomarkers, and cognitive staging. Relatively few, however, have analyzed plasma in relation to autopsy brain findings. Our study is the largest autopsy series to date, which allowed us to validate longitudinal plasma biomarkers against autopsy-confirmed diagnoses, examine specific associations with AD and other pathologies, and compare performance of plasma total tau, P-tau181 and P-tau231.

The influences of age and sex on plasma biomarkers have been analyzed in various cohorts. It may be difficult to distinguish aging effects from those of preclinical pathology (e.g., Aβ42). However, we found a significant effect of age on plasma Aβ42, a weak effect of age on plasma NfL, and weak effect of age on plasma P-tau181 and P-tau231 (age was included in our models). We found no significant effect of sex on any of the plasma biomarkers.

Several studies using immunoprecipitation-mass spectrometry (IP-MS) have shown that the ratio of plasma levels of Aβx-42/x-40 or Aβ1-42/1-40 [19, 28, 35, 40] is related to cerebral amyloidosis measured by PET, with AUCs from ROC analyses of approximately 0.7–0.8; this improved to 0.8 or higher in studies where age and *APOE* genotype were included in the algorithm. We obtained a lower AUC of 0.60 for plasma Aβ1-42/1-40 measured by a Simoa assay. This was similar to findings from another recent pathology series [7] and comparable to the Simoa results, but lower than IP-MS assay results, in a recent study that compared Simoa and IP-MS [18]. In the prior autopsy study and our study, Thal phase evaluation of amyloid pathology was available on a minority of subjects. Amyloid accumulation in diffuse plaques and cerebrovascular deposits occurs commonly in aging and this source contributes to the overall amyloid burden detectable by amyloid PET [14] and to Thal phase score [38]. Thus, it is not clear that Thal phase allows a more sensitive look into the relationship between amyloid and cognition or dementia than indices such as CERAD neuritic plaque density [41]. In CSF, levels of Aβ42 and Aβ42/40 appear to decrease before amyloid PET reaches significant thresholds [11, 23, 32], but it is challenging to determine thresholds and identify cutoff points for plasma Aβ42/40 which produces a much smaller effect size than CSF Aβ42/40 (difference between amyloid positive vs negative in relation to the overall distribution of values) [40]. This may be even more difficult among older people, many of whom harbor intermediate but subthreshold amyloid pathology. We found that plasma Aβ42/40 was lower in cases with significant amyloid pathology (moderate to severe by CERAD or in the limited set of patients, vs Thal phase), but with much overlap.

P-tau181 and P-tau231 performed comparably well in pathological comparisons of groups with or without AD neurofibrillary pathology, whereas total tau showed a small group increase only in people with severe (Braak stage VI) tau pathology. A previous study noted that P-tau231, measured using the same Simoa assay as in this study, showed high diagnostic value based on Tau PET, CSF amyloid and tau classification, and autopsy [3]. P-tau231 also showed increases earlier than P-tau181 and was able to discriminate Braak 0 vs Braak I–II stages. We had relatively few Braak 0 stage patients to allow this comparison, but still found that P-tau231 showed an earlier increase during intermediate stages of neuritic plaque pathology than P-tau181. Levels of P-tau231 and P-tau181 were correlated in our series, with the strength of correlation similar in AD (*r* = 0.69) and all cases (*r* = 0.68).

The timing of trajectories of changes in fragments of tau with different phospho-epitopes is of importance with regard to the potential to identify or track earlier stages in the development of AD. While levels of P-tau181, P-tau231 and P-tau217 are correlated in CSF [37], studies suggest that CSF P-tau217 and P-tau231 may show pathological changes earlier than P-tau181, and that CSF P-tau 217 correlates more strongly with amyloid and tau PET positivity than other forms of P-tau.[21]. Prior studies have identified relationships between plasma P-tau181 and amyloid PET positivity, but these relationships were weaker in cognitively normal people than in those with MCI or dementia [17, 25, 30]. P-tau231 was shown to be increased in plasma at lower levels of amyloid PET positivity than P-tau181, and in other studies increases in plasma P-tau217 appeared slightly earlier than in P-tau181 [25, 31]. In our study, longitudinal modeling of plasma P-tau231 and P-tau181 showed that both were significantly increased as early as 10 years before death in people who eventually showed clear AD pathology (i.e., NIA-Reagan High Likelihood) and continued to increase in the years leading up to death, and those levels were significantly higher than in people who died with low or intermediate AD pathology. These findings are similar to those recently reported for P-tau181 [20]; however, the baseline level of cognitive impairment in our cohort was milder (MMSE 22–23/30 in AD and 26/30 in people with low or no AD pathology) than in the previous study. We found that P-tau231 and P-tau181 had slightly different modeled trajectories vs CERAD neuritic plaque density, with P-tau231 showing a clearer continued increase compared to P-tau181 in people who eventually died with intermediate AD pathology. Previous studies showed that plasma P-tau231 showed increases as early as Braak II stage equivalent on tau PET scan [3]. Consistent with these findings, plasma P-tau231 was above threshold, to a greater extent than P-tau181, in some people in the Braak I–II subgroup in our autopsy series.

Even in analyses limited to individuals with normal cognition or MCI at baseline, higher baseline levels of plasma P-tau181 and P-tau231 (for analytical purposes, divided at the ROC cutoffs) predicted decline on tests of global cognition during 5 years of follow-up. This replicates clinical studies suggesting the potential use of plasma biomarkers, particularly forms of P-tau, as early predictive biomarkers of cognitive decline and dementia [8, 17, 33]. Although plasma NfL performed weakly relative to P-tau181 or 231 as a diagnostic biomarker, NfL levels above the cutoff predicted faster progression. Within our relatively small series, this suggests that there may be a subset of people with AD who have more intense neurodegeneration, reflected by NfL, which may, therefore, complement P-tau biomarkers as a predictor of cognitive decline. Further support for NfL as a predictor comes from data analyses from the BioFINDER study [8] and the Alzheimer’s Disease Neuroimaging Initiative (ADNI), where higher levels of plasma NfL predicted faster cognitive decline over 5–6 years in people with subtle cognitive changes or MCI [4]. Our study also suggests that cerebrovascular disease mechanisms or pathology may contribute to higher levels of plasma NfL and more rapid cognitive decline.

Multiple brain pathologies are common in AD and may be associated with faster progression of dementia [5, 16]. We found that plasma amyloid and P-tau biomarkers showed similar patterns in people with DLB or LATE-NC: no changes when pure DLB or LATE-NC were present, but increases consistent with an AD profile when significant concomitant AD was present. Plasma NfL was not helpful in pointing to either of these additional pathologies. In a recent large study across multiple cohorts [1], plasma NfL was helpful in detecting MCI and AD (with small to medium effect sizes) and disorders such as FTD, progressive supranuclear palsy and corticobasal syndrome, and was slightly increased in DLB [1]. In our series, the Low Pathology group had a mean plasma NfL level of 32 pg/mL, whereas in the multicenter study [1] an NfL level of 35 pg/mL was at the 90th percentile, and 38 pg/mL at the 95th percentile, when amyloid-negative controls were used to derive cutoffs. Our ROC-derived plasma NfL cutoff was 36.5 pg/mL, which has specificity of 90% (i.e., similar to the amyloid-negative control cutoffs in Ashton et al. [1]) and sensitivity of 42% for Braak stage VI AD. Plasma NfL sensitivity and specificity for this cutoff was higher for the small number of cases in our series with FTD pathology. NfL was slightly increased in association with HS pathology in the current study. Although this suggests that in an appropriate clinical setting a pattern of high plasma NfL and normal P-tau levels may point towards HS, there was too much overlap with ADNC to make this a useful discriminator.

Strengths of this study include the large number of subjects, standardization of clinical assessment, longitudinal plasma collection with harmonized preanalytical procedures and brain neuropathology assessment, and use of sensitive assays for plasma biomarkers. One limitation is the interval from last blood draw to autopsy: although this was relatively short, there is a gap of a few years during which brain pathology may progress, complicating the analysis of biomarker-pathology correlations. In addition, it is difficult in a study of this nature to obtain subjects with normal cognition who are free of brain pathology, especially covering a wide age range, therefore, we had limited capability to directly determine when plasma biomarkers might change, and our model of inflection points is less precise than similar models from large-scale clinical studies. Clinical studies have advantages over autopsy studies when establishing cutoffs or examining effects of age on biomarkers, but for some questions they may be confounded by unknown co-pathologies.

This study provides support for plasma biomarkers in relation to diagnosis and prognosis based on the ATN framework. The biomarkers measured in our study do not identify other common pathologies that may occur on their own or together with AD pathology and may contribute to dementia, in particular a-synuclein and TDP-43 pathology. A potential biochemical marker for detecting DLB is α-synuclein. RT-QuIC measures that detect α-synuclein aggregates capable of seeding fibril formation have high accuracy when measured in CSF or skin biopsy samples but are not yet detectable in plasma [10, 24, 34]. Unfortunately, no specific biochemical marker for HS or LATE is available at present. The great progress that has been made in plasma biomarkers for ATN raises hope for developing a more extensive panel of plasma biomarkers to comprehensively profile other common brain pathologies in relation to cognitive decline.

## Supplementary Information

Below is the link to the electronic supplementary material.

Supplementary file1 (DOCX 892 kb)
