# Supplementary material for: Plasma biomarkers for Alzheimer’s Disease in relation to neuropathology and cognitive change
Source: Acta Neuropathol. 2022 Feb 23;143(4):487–503. doi: 10.1007/s00401-022-02408-5 (PMC8960664; doi:10.1007/s00401-022-02408-5)
Supplement: Supplementary file 1 — Supplementary file1 (DOCX 892 kb) [file 401_2022_2408_MOESM1_ESM.docx]

**Supplementary Tables and Figures, On-line Resources**

On-line Resource Table 1: Plasma Biomarkers at Last Blood Draw

|  | Low Pathology | Intermediate ADNC | High ADNC | Other Pathology | Intermediate ADNC + Other | High ADNC  + Other |
| --- | --- | --- | --- | --- | --- | --- |
| n | 29 | 19 | 124 | 45 | 29 | 66 |
| Aβ42 | 14.2 ± 4.7 | 14.4 ± 5.8 | 12.1 ± 4.4 | 13.9 ± 4 | 13.1 ± 5.9 | 12 ± 3.5 |
| Aβ40 | 247.7 ± 75.2 | 288.2 ± 83.6 | 216 ± 64.1 | 233.7 ± 66.5 | 219.5 ± 84.8 | 218.2 ± 56.8 |
| Aβ42/Aβ40 | 0.058 ± 0.013 | 0.050 ± 0.014 | 0.057 ± 0.014 | 0.060 ± 0.012 | 0.062 ± 0.016 | 0.055 ± 0.011 |
| T-tau | 2.2 ± 1.1 | 2.4 ± 1.2 | 2.3 ± 1.4 | 1.9 ± 1.1 | 1.7 ± 0.7 | 2.2 ± 1.1 |
| P-tau181 | 3.3 ± 1.5 | 4.2 ± 2.9 | 5.4 ± 2.2 | 3.9 ± 1.8 | 3.9 ± 1.8 | 5.4 ± 2.3 |
| P-tau231 | 8.9 ± 3.8 | 11.7 ± 5.3 | 13.3 ± 5.1 | 10.1 ± 4.9 | 11 ± 5.6 | 14 ± 5.1 |
| NfL | 32.3 ± 13.8 | 44 ± 31.6 | 39.3 ± 24.5 | 67.3 ± 167.4 | 40.5 ± 41.4 | 37.3 ± 22.3 |

Values of biomarkers are in pg/mL. On-line Resource Supplementary Table 2 contains statistical comparisons

On-line Resource Table 2. Statistical comparisons for plasma biomarkers in different pathologically-defined groups

|  | AB42/AB40 | | | T-tau | | | P-tau181 | | | P-tau231 | | | NfL | | |
| --- | --- | --- | --- | --- | --- | --- | --- | --- | --- | --- | --- | --- | --- | --- | --- |
| contrast | Estimate (SE) | Raw P value | Adj P  value | Estimate (SE) | Raw P value | Adj P  value | Estimate (SE) | Raw P value | Adj P  value | Estimate (SE) | Raw P value | Adj P  value | Estimate (SE) | Raw P value | Adj P  value |
| Low Path - Intermediate ADNC | 0.0077 (0.0037) | 0.042 | 0.320 | -0.31 (0.36) | 0.383 | 0.952 | -0.95 (0.63) | 0.136 | 0.669 | -2.67 (1.48) | 0.072 | 0.465 | -14.28 (19.74) | 0.470 | 0.979 |
| Low Path - High ADNC | 0.0033 (0.0028) | 0.236 | 0.843 | -0.23 (0.27) | 0.388 | 0.955 | **-2.22 (0.47)** | **3.6 x 10^-6^** | **5.2 x 10^-5^** | **-4.83 (1.1)** | **1.5 x 10^-5^** | **2.2 x 10^-4^** | -13.02 (14.5) | 0.370 | 0.947 |
| Low Path - Other Path | -0.0019 (0.0031) | 0.544 | 0.990 | 0.25 (0.31) | 0.411 | 0.963 | -0.54 (0.53) | 0.309 | 0.911 | -1.17 (1.23) | 0.344 | 0.934 | -40.31 (16.36) | 0.014 | 0.139 |
| Low Path - (Intermediate ADNC + Other) | -0.0034 (0.0034) | 0.319 | 0.918 | 0.65 (0.33) | 0.052 | 0.372 | -0.58 (0.57) | 0.316 | 0.916 | -1.98 (1.34) | 0.141 | 0.679 | -5.69 (17.78) | 0.749 | 0.999 |
| Low Path - (High ADNC + Other) | 0.004 (0.0029) | 0.171 | 0.743 | -0.14 (0.29) | 0.628 | 0.997 | **-2.24 (0.5)** | **9.9 x 10^-6^** | **1.4 x 10^-4^** | **-5.33 (1.17)** | **7.3 x 10^-6^** | **1.0 x 10^-4^** | -13.08 (15.4) | 0.397 | 0.958 |
| Intermediate ADNC - High ADNC | -0.0044 (0.0033) | 0.182 | 0.763 | 0.08 (0.31) | 0.799 | 0.999 | -1.27 (0.55) | 0.022 | 0.198 | -2.16 (1.29) | 0.096 | 0.552 | 1.27 (17.28) | 0.942 | 0.999 |
| Intermediate ADNC - Other Path | -0.0095 (0.0036) | 0.008 | 0.083 | 0.57 (0.34) | 0.096 | 0.552 | 0.41 (0.6) | 0.503 | 0.985 | 1.5 (1.4) | 0.284 | 0.892 | -26.02 (18.8) | 0.167 | 0.737 |
| Intermediate ADNC - (Intermediate ADNC + Other) | **-0.011 (0.0038)** | **0.004** | **0.047** | 0.97 (0.37) | 0.009 | 0.091 | 0.37 (0.64) | 0.563 | 0.992 | 0.7 (1.5) | 0.642 | 0.997 | 8.59 (20.1) | 0.669 | 0.998 |
| Intermediate ADNC - (High ADNC + Other) | -0.0036 (0.0034) | 0.286 | 0.893 | 0.18 (0.32) | 0.583 | 0.994 | -1.29 (0.57) | 0.025 | 0.220 | -2.65 (1.34) | 0.049 | 0.359 | 1.21 (17.97) | 0.946 | 0.999 |
| High ADNC - Other Path | -0.0052 (0.0023) | 0.023 | 0.204 | 0.49 (0.22) | 0.027 | 0.232 | **1.68 (0.39)** | **2.1 x 10^-5^** | **3.0 x 10^-4^** | **3.66 (0.89)** | **5.3 x 10^-5^** | **0.001** | -27.29 (11.89) | 0.022 | 0.199 |
| High ADNC - (Intermediate ADNC + Other) | -0.0066 (0.0027) | 0.014 | 0.138 | **0.89 (0.27)** | **0.001** | **0.012** | **1.64 (0.45)** | **3.5 x 10^-4^** | **0.005** | 2.86 (1.06) | 0.008 | 0.080 | 7.33 (14.18) | 0.606 | 0.996 |
| High ADNC - (High ADNC + Other) | 0.0007 (0.0019) | 0.702 | 0.999 | 0.1 (0.18) | 0.600 | 0.995 | -0.02 (0.33) | 0.947 | 0.999 | -0.49 (0.78) | 0.526 | 0.988 | -0.06 (10.25) | 0.995 | 0.999 |
| Other Path - (Intermediate ADNC + Other) | -0.0015 (0.003) | 0.627 | 0.997 | 0.4 (0.3) | 0.182 | 0.764 | -0.03 (0.52) | 0.949 | 0.999 | -0.81 (1.2) | 0.501 | 0.985 | 34.62 (16.06) | 0.032 | 0.262 |
| Other Path - (High ADNC + Other) | 0.0059 (0.0025) | 0.018 | 0.164 | -0.39 (0.24) | 0.101 | 0.568 | **-1.7 (0.42)** | **7.8 x 10^-5^** | **0.001** | **-4.16 (0.98)** | **2.8 x 10^-5^** | **4.0 x 10^-4^** | 27.23 (13.02) | 0.037 | 0.295 |
| (Intermediate ADNC + Other) - (High ADNC + Other) | 0.0074 (0.0029) | 0.011 | 0.111 | -0.79 (0.28) | 0.005 | 0.060 | **-1.66 (0.49)** | **0.001** | **0.009** | **-3.35 (1.14)** | **0.004** | **0.041** | -7.39 (15.21) | 0.628 | 0.997 |

Effect sizes and both raw and multiple-comparisons adjusted p-values for pairwise comparisons of plasma biomarkers between diagnostic groups. Statistics are corrected for multiple comparisons using Tukey’s method to maintain a family error rate of 0.05, with values reaching this significance threshold bolded. A graphical representation of this data is available in Figure 1.

On-line Resource Figure 1. Plasma biomarkers by diffuse plaque density and amyloid angiopathy

Boxplots of the distributions of the plasma biomarkers from the blood draw closest to death in individuals with various degrees of (a) diffuse amyloid plaques (b) amyloid angiopathy. One NfL value of 1154 in FTLD patient was removed from plots for visualization, but was retained in statistical analyses. Effect sizes and both raw and multiple-comparisons adjusted p-values are available in Supplementary Table 6, On-line Resource. Statistics for pairwise comparisons are corrected for multiple comparisons using Tukey’s method to maintain a family error rate of 0.05, and are graphically summarized as follows:

* p<.05, ** p<.01, *** p<.001


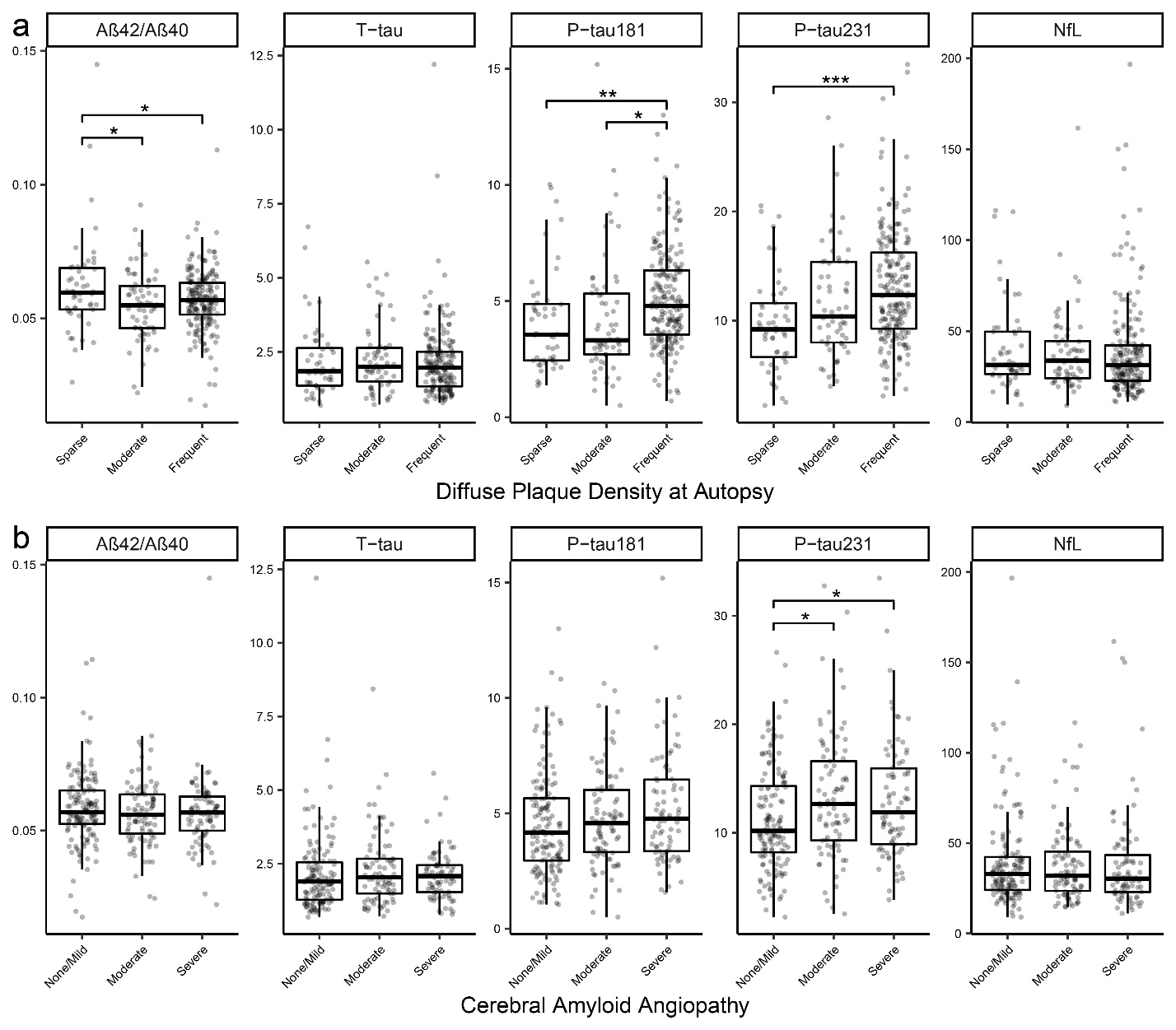


On-line resource Table 3. Statistical comparisons for plasma biomarkers in relation to diffuse amyloid plaques and amyloid angiopathy

| 1. **Diffuse Plaque Density** | | | | | | | | | | | | | | | | |
| --- | --- | --- | --- | --- | --- | --- | --- | --- | --- | --- | --- | --- | --- | --- | --- | --- |
|  | AB42/AB40 | | | T-tau | | | P-tau181 | | | P-tau231 | | | NfL | | |  |
| contrast | Estimate (SE) | Raw P value | Adj P  value | Estimate (SE) | Raw P value | Adj P  value | Estimate (SE) | Raw P value | Adj P  value | Estimate (SE) | Raw P value | Adj P  value | Estimate (SE) | Raw P value | Adj P  value |  |
| Sparse - Moderate | **0.0068 (0.0024)** | **0.005** | **0.015** | -0.02 (0.24) | 0.941 | 0.997 | -0.17 (0.43) | 0.700 | 0.922 | -2.14 (0.98) | 0.031 | 0.078 | -9.4 (12.76) | 0.462 | 0.742 |  |
| Sparse - Frequent | **0.0049 (0.002)** | **0.017** | **0.044** | 0.04 (0.2) | 0.848 | 0.980 | **-1.08 (0.36)** | **0.003** | **0.008** | **-3.49 (0.82)** | **2.6 x 10^-5^** | **7.6 x 10^-5^** | 3.41 (10.69) | 0.75 | 0.945 |  |
| Moderate - Frequent | -0.0019 (0.0019) | 0.299 | 0.552 | 0.06 (0.18) | 0.758 | 0.949 | **-0.91 (0.33)** | **0.006** | **0.015** | -1.36 (0.76) | 0.074 | 0.174 | 12.81 (9.71) | 0.188 | 0.385 |  |

| 1. **Cerebral Amyloid Angiopathy** | | | | | | | | | | | | | | | | |
| --- | --- | --- | --- | --- | --- | --- | --- | --- | --- | --- | --- | --- | --- | --- | --- | --- |
|  | AB42/AB40 | | | T-tau | | | P-tau181 | | | P-tau231 | | | NfL | | | |
| contrast | Estimate (SE) | Raw P value | Adj P  value | Estimate (SE) | Raw P value | Adj P  value | Estimate (SE) | Raw P value | Adj P  value | Estimate (SE) | Raw P value | Adj P  value | Estimate (SE) | Raw P value | Adj P  value |  |
| None/Mild - Moderate | 0.0019 (0.0018) | 0.278 | 0.523 | -0.23 (0.17) | 0.184 | 0.378 | -0.22 (0.31) | 0.479 | 0.758 | **-1.98 (0.72)** | **0.007** | **0.018** | 4.08 (9.24) | 0.659 | 0.898 |  |
| None/Mild - Severe | 0.0016 (0.0018) | 0.371 | 0.644 | -0.08 (0.18) | 0.637 | 0.884 | -0.69 (0.33) | 0.034 | 0.085 | **-1.77 (0.75)** | **0.018** | **0.048** | 2.22 (9.55) | 0.816 | 0.971 |  |
| Moderate - Severe | -3e-04 (0.002) | 0.883 | 0.988 | 0.14 (0.19) | 0.460 | 0.74 | -0.47 (0.35) | 0.182 | 0.375 | 0.21 (0.82) | 0.799 | 0.965 | -1.85 (10.41) | 0.859 | 0.983 |  |

Effect sizes and both raw and multiple-comparisons adjusted p-values for pairwise comparisons of plasma biomarkers between pathologically-defined groups. Statistics are corrected for multiple comparisons using Tukey’s method to maintain a family error rate of 0.05, with values reaching this significance threshold bolded. A graphical representation of this data is available in Supplementary Figure 1, On-line Resource.

On line resource Table 4: Statistical comparisons of plasma biomarkers in groups defined by different staging approaches to ADNC

| 1. **CERAD Neuritic Plaque Density** | | | | | | | | | | | | | | | | | | | | | | | | | | | | | |  |
| --- | --- | --- | --- | --- | --- | --- | --- | --- | --- | --- | --- | --- | --- | --- | --- | --- | --- | --- | --- | --- | --- | --- | --- | --- | --- | --- | --- | --- | --- | --- |
|  | AB42/AB40 | | | | | | T-tau | | | | | P-tau181 | | | | | | P-tau231 | | | | | | NfL | | | | | |  |
| contrast | Estimate (SE) | | | Raw P value | Adj P  value | | Estimate (SE) | | Raw P value | Adj P  value | | Estimate (SE) | | Raw P value | | Adj P  value | | Estimate (SE) | | Raw P value | | Adj P  value | | Estimate (SE) | | Raw P value | Adj P  value | |  |  |
| Sparse - Moderate | **0.0064 (0.0023)** | | | 0.005 | **0.014** | | -0.1 (0.22) | | 0.645 | 0.889 | | -0.91 (0.39) | | 0.021 | | 0.054 | | **-3.25 (0.91)** | | **4.0 x 10^-4^** | | **0.001** | | -7.77 (11.92) | | 0.515 | 0.792 | |  |  |
| Sparse - Frequent | **0.0054 (0.0022)** | | | 0.018 | **0.046** | | -0.3 (0.22) | | 0.170 | 0.355 | | **-1.99 (0.39)** | | **6.1 x 10^-7^** | | **1.8 x 10^-6^** | | **-4.14 (0.9)** | | **6.3 x 10^-6^** | | **1.9 x 10^-5^** | | 3.04 (11.83) | | 0.797 | 0.964 | |  |  |
| Moderate - Frequent | -0.001 (0.0017) | | | 0.547 | 0.819 | | -0.2 (0.17) | | 0.227 | 0.448 | | **-1.07 (0.29)** | | **2.6 x 10^-4^** | | **7.7 x 10^-4^** | | -0.9 (0.69) | | 0.192 | | 0.393 | | 10.81 (8.96) | | 0.228 | 0.450 | |  |  |
| 1. **Braak Stage** | | | | | | | | | | | | | | | | | | | | | | | | | | | | | | |
|  | AB42/AB40 | | | | | | T-tau | | | | | P-tau181 | | | | | | P-tau231 | | | | | | NfL | | | | | | |
| contrast | Estimate (SE) | | | Raw P value | Adj P  value | | Estimate (SE) | | Raw P value | Adj P  value | | Estimate (SE) | | Raw P value | | Adj P  value | | Estimate (SE) | | Raw P value | | Adj P  value | | Estimate (SE) | | Raw P value | Adj P  value | |  |  |
| (0 - II) - (III - IV) | 9e-04 (0.0024) | | | 0.700 | 0.921 | | 0.13 (0.23) | | 0.576 | 0.841 | | -0.55 (0.39) | | 0.167 | | 0.350 | | -1.22 (0.92) | | 0.185 | | 0.380 | | 15.83 (12.35) | | 0.201 | 0.406 | |  |  |
| (0 - II) - (V - VI) | **0.0044 (0.0019)** | | | **0.019** | **0.050** | | -0.35 (0.18) | | 0.061 | 0.146 | | **-1.98 (0.32)** | | **1.8 x 10^-9^** | | **5.5 x 10^-9^** | | **-4.23 (0.74)** | | **2.6 x 10^-8^** | | **7.7 x 10^-8^** | | 13.46 (9.88) | | 0.174 | 0.362 | |  |  |
| (III - IV) - (V - VI) | 0.0035 (0.0021) | | | 0.089 | 0.204 | | **-0.47 (0.2)** | | **0.018** | **0.047** | | **-1.43 (0.34)** | | **4.2 x 10^-5^** | | **1.2 x 10^-4^** | | **-3.01 (0.81)** | | **2.5 x 10^-4^** | | **6.9 x 10^-4^** | | -2.36 (10.86) | | 0.828 | 0.974 | |  |  |
| 1. **NIA-Reagan Criteria for ADNC** | | | | | | | | | | | | | | | | | | | | | | | | | | | | | |  |
|  | | AB42/AB40 | | | | | | T-tau | | | | | P-tau181 | | | | | | P-tau231 | | | | | | NfL | | | | |  |
| contrast | | Estimate (SE) | Raw P value | | | Adj P  value | | Estimate (SE) | Raw P value | | Adj P  value | | Estimate (SE) | | Raw P value | | Adj P  value | | Estimate (SE) | | Raw P value | | Adj P  value | | Estimate (SE) | Raw P value | Adj P  value |  |  |  |
| Low ADNC - Intermediate ADNC | | 0.0021 (0.0024) | 0.383 | | | 0.657 | | 0.1 (0.23) | 0.677 | | 0.909 | | -0.4 (0.4) | | 0.316 | | 0.575 | | -1.55 (0.93) | | 0.096 | | 0.219 | | 14.83 (12.5) | 0.236 | 0.462 |  |  |  |
| Low ADNC - High ADNC | | **0.0048 (0.0018)** | **0.008** | | | **0.022** | | -0.37 (0.18) | 0.039 | | 0.097 | | **-1.89 (0.31)** | | **2.6 x 10^-9^** | | **7.8 x 10^-9^** | | **-4.28 (0.71)** | | **5.4 x 10^-9^** | | **1.6 x 10^-8^** | | 12.17 (9.55) | 0.203 | 0.410 |  |  |  |
| Intermediate ADNC - High ADNC | | 0.0028 (0.0022) | 0.206 | | | 0.414 | | -0.46 (0.21) | 0.028 | | 0.072 | | **-1.49 (0.36)** | | **5.0 x 10^-5^** | | **1.5 x 10^-4^** | | **-2.73 (0.85)** | | **0.001** | | **0.004** | | -2.66 (11.4) | 0.816 | 0.970 |  |  |  |

Effect sizes and both raw and multiple-comparisons adjusted p-values for pairwise comparisons of plasma biomarkers between pathologically-defined groups. Statistics are corrected for multiple comparisons using Tukey’s method to maintain a family error rate of 0.05, with values reaching this significance threshold bolded. A graphical representation of this data is available in Figure 2.

On-line resource Table 5. Statistical comparisons for plasma biomarkers in relation to Hippocampal sclerosis (HS), Limbic Age-related TDP-43 Encephalopathy (LATE), Lewy Body Disease (LBD) and ADNC

| 1. **Hippocampal Sclerosis of Aging (HSA)** | | | | | | | | | | | | | | | | | | | | | | | | | | | | | |  |
| --- | --- | --- | --- | --- | --- | --- | --- | --- | --- | --- | --- | --- | --- | --- | --- | --- | --- | --- | --- | --- | --- | --- | --- | --- | --- | --- | --- | --- | --- | --- |
|  | AB42/AB40 | | | | | | T-tau | | | | | P-tau181 | | | | | | P-tau231 | | | | | | NfL | | | | | |  |
| contrast | Estimate (SE) | | | Raw P value | Adj P  value | | Estimate (SE) | | Raw P value | Adj P  value | | Estimate (SE) | | Raw P value | | Adj P  value | | Estimate (SE) | | Raw P value | | Adj P  value | | Estimate (SE) | | Raw P value | Adj P  value | |  |  |
| ADNC - (ADNC + HS) | -7e-04 (0.0025) | | | 0.776 | 0.956 | | -0.15 (0.26) | | 0.556 | 0.826 | | 0.58 (0.46) | | 0.210 | | 0.421 | | -0.11 (1.07) | | 0.922 | | 0.995 | | **-13.93 (5.26)** | | **0.018** | **0.050** | |  |  |
| ADNC - HS | -0.0038 (0.0028) | | | 0.173 | 0.360 | | 0.15 (0.3) | | 0.624 | 0.875 | | **1.82 (0.5)** | | **3.6 x 10^-4^** | | **0.001** | | **3.33 (1.15)** | | **0.004** | | **0.012** | | **-15.36 (4.43)** | | **0.001** | **0.002** | |  |  |
| (ADNC + HS) - HS | -0.0031 (0.0034) | | | 0.369 | 0.640 | | 0.3 (0.36) | | 0.409 | 0.686 | | 1.24 (0.62) | | 0.048 | | 0.116 | | **3.43 (1.44)** | | **0.018** | | **0.048** | | -2.83 (4.12) | | 0.532 | 0.791 | |  |  |
| 1. **Hippocampal TDP-43 Positivity (LATE)** | | | | | | | | | | | | | | | | | | | | | | | | | | | | | | |
|  | AB42/AB40 | | | | | | T-tau | | | | | P-tau181 | | | | | | P-tau231 | | | | | | NfL | | | | | | |
| contrast | Estimate (SE) | | | Raw P value | Adj P  value | | Estimate (SE) | | Raw P value | Adj P  value | | Estimate (SE) | | Raw P value | | Adj P  value | | Estimate (SE) | | Raw P value | | Adj P  value | | Estimate (SE) | | Raw P value | Adj P  value | |  |  |
| ADNC - (ADNC + TDP-43) | -8e-04 (0.002) | | | 0.687 | 0.914 | | 0.06 (0.33) | | 0.862 | 0.983 | | 0.48 (0.46) | | 0.298 | | 0.55 | | -0.71 (1.04) | | 0.495 | | 0.773 | | -2.22 (5.98) | | 0.711 | 0.927 | |  |  |
| ADNC - (TDP-43) | -0.0016 (0.0026) | | | 0.549 | 0.819 | | 0.3 (0.45) | | 0.509 | 0.786 | | **2.43 (0.59)** | | **7.5 x 10^-5^** | | **0.0002** | | **4.73 (1.3)** | | **4.1 x 10^-4^** | | **0.001** | | -7.09 (7.72) | | 0.360 | 0.630 | |  |  |
| (ADNC + TDP-43) - (TDP-43) | -8e-04 (0.0029) | | | 0.795 | 0.963 | | 0.24 (0.49) | | 0.624 | 0.876 | | **1.94 (0.66)** | | **0.004** | | **0.011** | | **5.44 (1.45)** | | **2.8 x 10^-4^** | | **0.001** | | -4.87 (8.56) | | 0.571 | 0.837 | |  |  |
| 1. **Lewy Body Disease (LBD)** | | | | | | | | | | | | | | | | | | | | | | | | | | | | | |  |
|  | | AB42/AB40 | | | | | | T-tau | | | | | P-tau181 | | | | | | P-tau231 | | | | | | NfL | | | | |  |
| contrast | | Estimate (SE) | Raw P value | | | Adj P  value | | Estimate (SE) | Raw P value | | Adj P  value | | Estimate (SE) | | Raw P value | | Adj P  value | | Estimate (SE) | | Raw P value | | Adj P  value | | Estimate (SE) | Raw P value | Adj P  value |  |  |  |
| ADNC - (ADNC + LBD) | | 0.0011 (0.0023) | 0.65 | | | 0.893 | | 0.19 (0.23) | 0.401 | | 0.677 | | -0.17 (0.4) | | 0.679 | | 0.910 | | -0.23 (0.97) | | 0.815 | | 0.970 | | 8.28 (3.97) | 0.038 | 0.095 |  |  |  |
| ADNC - LBD | | **-0.0065 (0.0023)** | **0.005** | | | **0.013** | | **0.84 (0.23)** | **3.4 x 10^-4^** | | **0.001** | | **1.33 (0.4)** | | **0.001** | | **0.003** | | **2.93 (0.94)** | | **0.002** | | **0.006** | | **11.51 (3.88)** | **0.003** | **0.009** |  |  |  |
| (ADNC + LBD) - LBD | | **-0.0075 (0.0028)** | **0.009** | | | **0.024** | | 0.66 (0.28) | 0.022 | | 0.056 | | **1.49 (0.5)** | | **0.003** | | **0.009** | | **3.16 (1.19)** | | **0.009** | | **0.023** | | 3.23 (4.87) | 0.508 | 0.785 |  |  |  |

Effect sizes and both raw and multiple-comparisons adjusted p-values for pairwise comparisons of plasma biomarkers between pathologically-defined groups. Statistics are corrected for multiple comparisons using Tukey’s method to maintain a family error rate of 0.05, with values reaching this significance threshold bolded. A graphical representation of this data is available in Figure 3.

**On-line Resource Table 6.** Cohorts with LATE-NC staged as present or absent in the hippocampus using TDP43 immunohistochemistry

| variable | ADNC | ADNC + TDP-43 | TDP-43 |
| --- | --- | --- | --- |
| n | 73 | 29 | 14 |
| Age at Baseline | 74.7 ± 9.6 | 80.1 ± 5 | 80.3 ± 10.6 |
| Age at Last Plasma | 77.4 ± 10.3 | 83 ± 5.2 | 82.5 ± 10.3 |
| Age at Death | 79.8 ± 10.2 | 85.6 ± 5.5 | 84.5 ± 9.9 |
| Last Plasma - Death (years) | 2.4 ± 1.2 | 2.6 ± 1.5 | 2 ± 1.1 |
| Female | 30 (41%) | 7 (24%) | 5 (36%) |
| Hispanic | 4 (5%) | 1 (4%) | 1 (7%) |
| EDUC | 14.9 ± 3.4 | 16.2 ± 2 | 14.4 ± 3.5 |
| APOE 0 e4 alleles | 33 (45%) | 10 (34%) | 9 (64%) |
| APOE 1 e4 allele | 30 (41%) | 18 (62%) | 4 (29%) |
| APOE 2 e4 alleles | 10 (14%) | 1 (3%) | 1 (7%) |
| Baseline MMSE | 22.3 ± 5.9 | 21.4 ± 5.4 | 20.9 ± 6.7 |
| Baseline DRS | 109.5 ± 25.8 | 111.6 ± 16.9 | 109.1 ± 22.4 |
| Baseline CDR-sob | 6.5 ± 4 | 6.1 ± 3.2 | 6 ± 5.2 |
| Final MMSE | 15.6 ± 7.1 | 17.1 ± 6.2 | 19.1 ± 5.5 |
| Final DRS | 86.5 ± 29.8 | 93.4 ± 23.9 | 97.6 ± 25.4 |
| Final CDR-sob | 11.2 ± 4.5 | 10.4 ± 4.3 | 8.4 ± 5.8 |
| Final Clinical DX: Normal | 1 (1%) | 0 (0%) | 0 (0%) |
| Final Clinical DX: MCI | 1 (1%) | 1 (3%) | 1 (7%) |
| Final Clinical Dx: AD | 63 (86%) | 26 (90%) | 10 (71%) |
| Final Clinical DX: DLB/PDD | 6 (8%) | 2 (7%) | 3 (21%) |
| Final Clinical DX: FTLD | 1 (1%) | 0 (0%) | 0 (0%) |
| Final Clinical DX: Other | 1 (1%) | 0 (0%) | 0 (0%) |
| # Blood Samples | 2.6 ± 1.5 | 2.4 ± 1.1 | 2.1 ± 1.5 |
| # Clinical Visits | 4.6 ± 2.3 | 5.3 ± 2.3 | 6 ± 2.9 |
| Final Plasma Aβ42 | 12.1 ± 4 | 12.3 ± 2.9 | 12.8 ± 3.3 |
| Final Plasma Aβ40 | 223.1 ± 59.2 | 221.4 ± 57.9 | 229.2 ± 60.7 |
| Final Plasma Aβ42/Aβ40 | 0.055 ± 0.009 | 0.057 ± 0.008 | 0.057 ± 0.008 |
| Final Plasma T-tau | 2.4 ± 1.5 | 2.2 ± 1.4 | 2.2 ± 1.3 |
| Final Plasma P-tau181 | 5.5 ± 2.2 | 5.1 ± 1.7 | 3.2 ± 1.2 |
| Final Plasma P-tau231 | 13.5 ± 4.4 | 14.4 ± 4.6 | 8.9 ± 2.7 |
| Final Plasma NfL | 38.2 ± 24.8 | 41.8 ± 26.6 | 49.5 ± 37.3 |

**On-line resource Figure 2**. Longitudinal changes of plasma biomarkers in relation to ADNC without concomitant pathologies

Longitudinal progression in biomarkers in the 10 years prior to death in study participants divided by their degree of ADNC after excluding concomitant neuropathologies including FTLD, HS, LBD, and other significant pathologies. Horizontal dashed lines represent the thresholds derived from ROC analyses presented in Figure 4. Thick lines represent predictions of the trajectories of the biomarkers for an demographically average participant, derived from mixed effects models with covariates added for age, sex, interval from last visit to death, as well as each variable’s interaction with time. All models included random intercepts and slopes by participant.


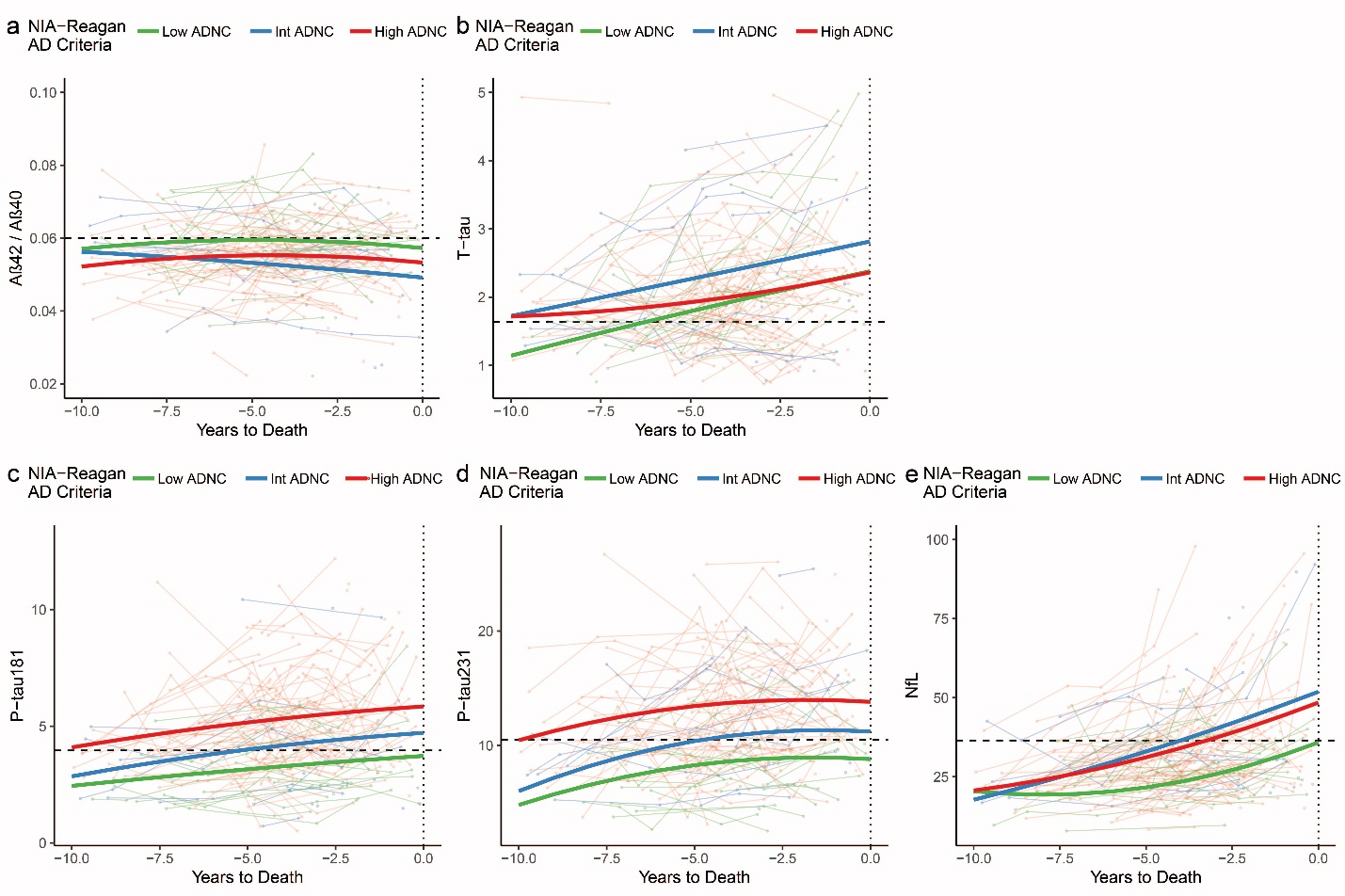


**On-line Resource Figure 3:** ROC analyses of a subset of cases with concurrent structural T1-weighted MRI.

ROC curves and associated thresholds, specificities, sensitivities, and areas under the curve (AUCs) for the use of each plasma biomarker, structural MRI measure, or their combination to distinguish patients who were classified as high ADNC at autopsy vs those that were not in a subset of 76 individuals with structural T1-weighted MRI obtained within 2 years of the plasma biomarker measurement. The average hippocampal volume was corrected for the estimated total intracranial volume (eTIV). When compared to the AUCs of the single variable curves using DeLong’s test, Model 1 (combining entorhinal thickness and P-tau181) performed better than entorhinal thickness alone (p=0.004), but comparable to P-tau181 alone (p=0.37).


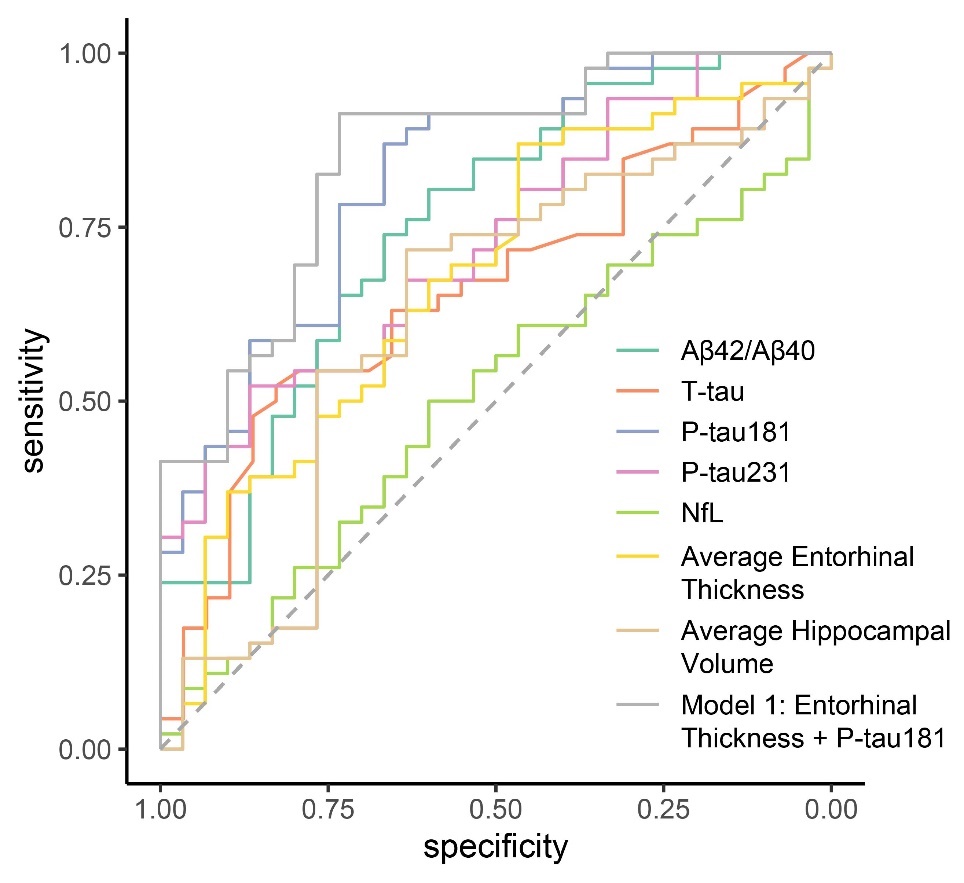


|  | **threshold** | **specificity** | **sensitivity** | **AUC** |
| --- | --- | --- | --- | --- |
| **Aβ42/Aβ40** | 0.0583 | 0.6667 | 0.7391 | 0.7507 |
| **T-tau** | 1.9650 | 0.8276 | 0.5217 | 0.6623 |
| **P-tau181** | 3.3042 | 0.6667 | 0.8696 | 0.8203 |
| **P-tau231** | 13.4179 | 0.8667 | 0.5217 | 0.7290 |
| **NfL** | 28.6823 | 0.6000 | 0.5000 | 0.5058 |
| **Average Entorhinal Thickness** | 3.0913 | 0.4667 | 0.8696 | 0.6772 |
| **Average Hippocampal Volume (eTIV corrected)** | 2.0637 | 0.6333 | 0.7174 | 0.6261 |
| **Model 1: Entorhinal Thickness + P-tau181** | 0.4651 | 0.7333 | 0.9130 | 0.8493 |
